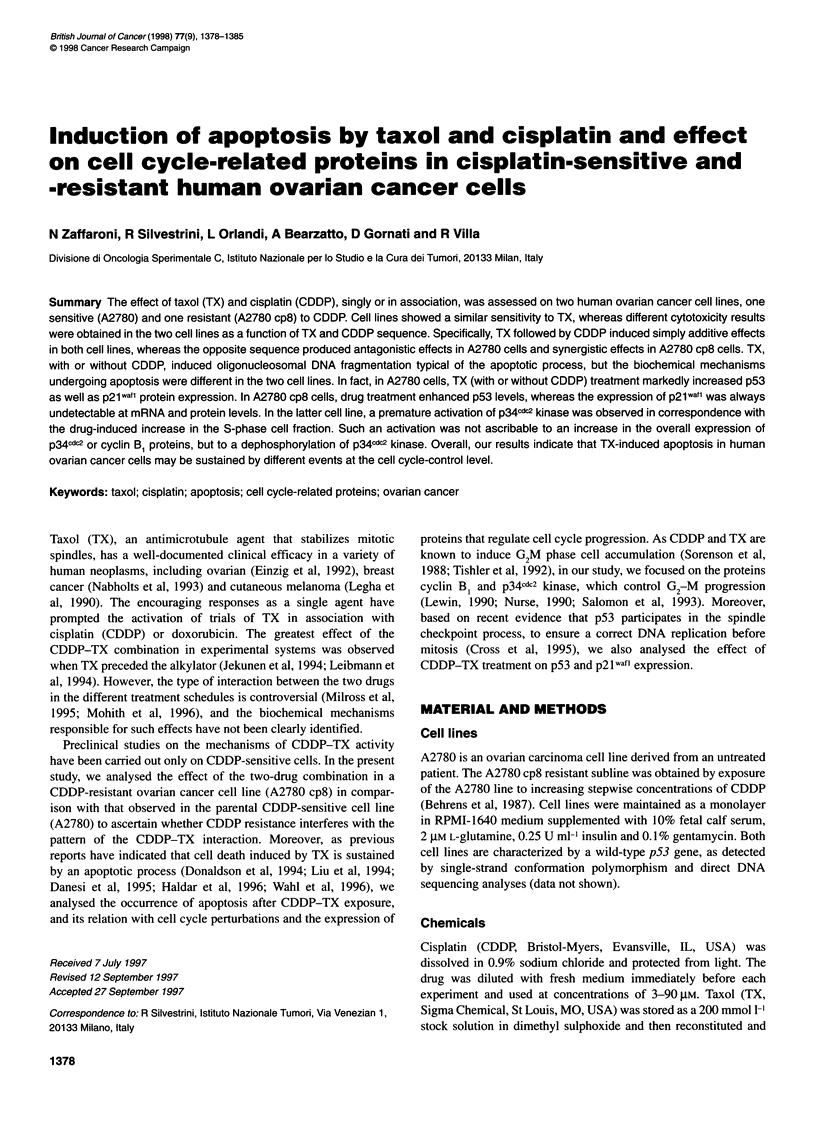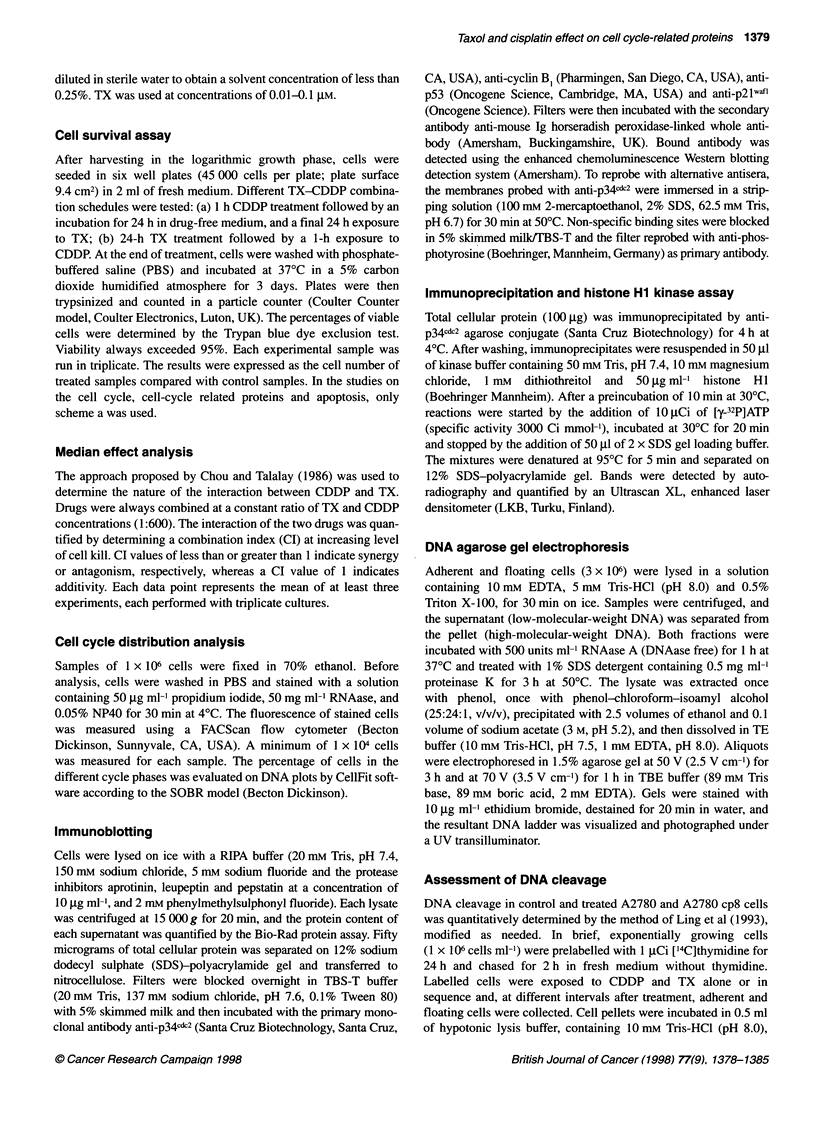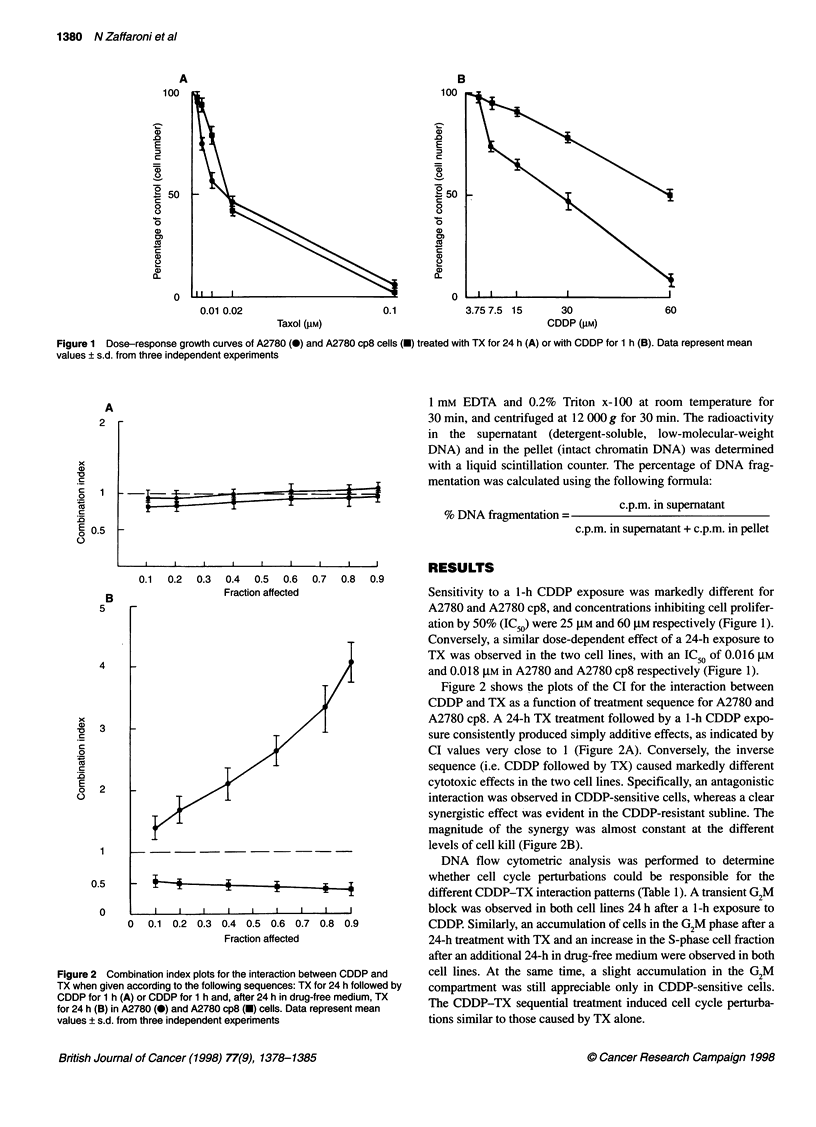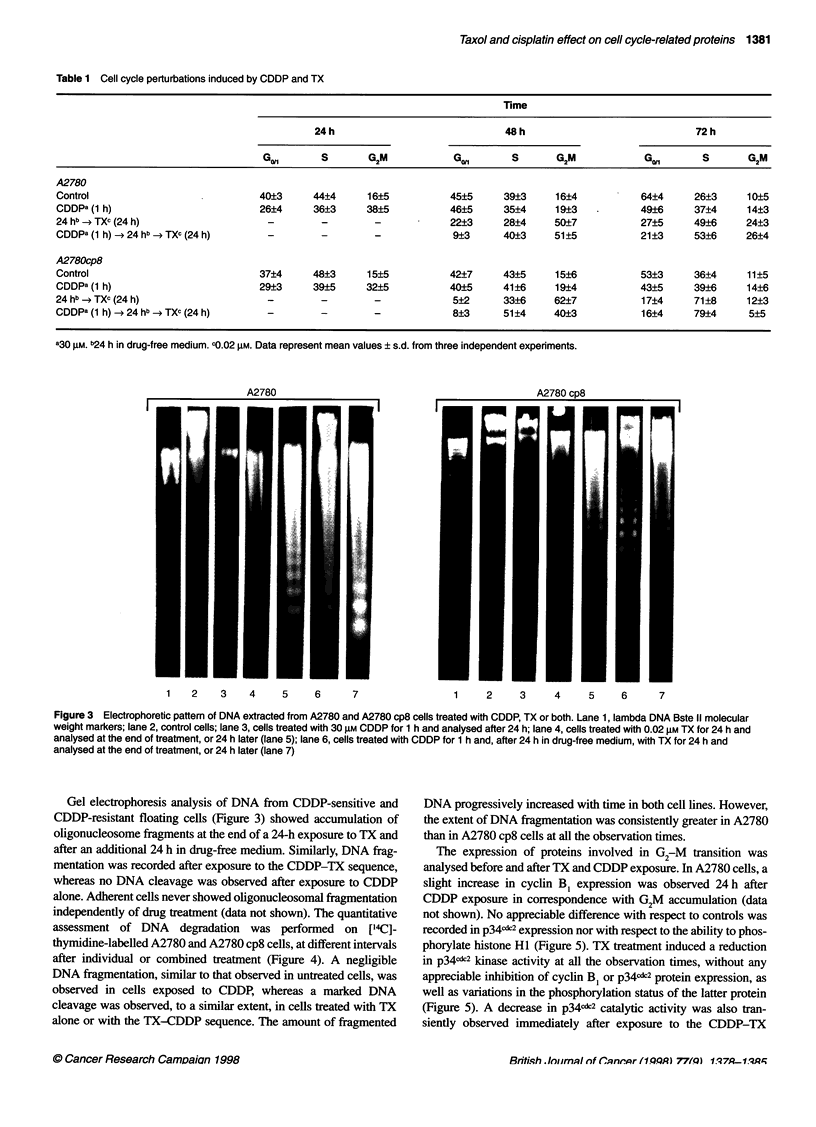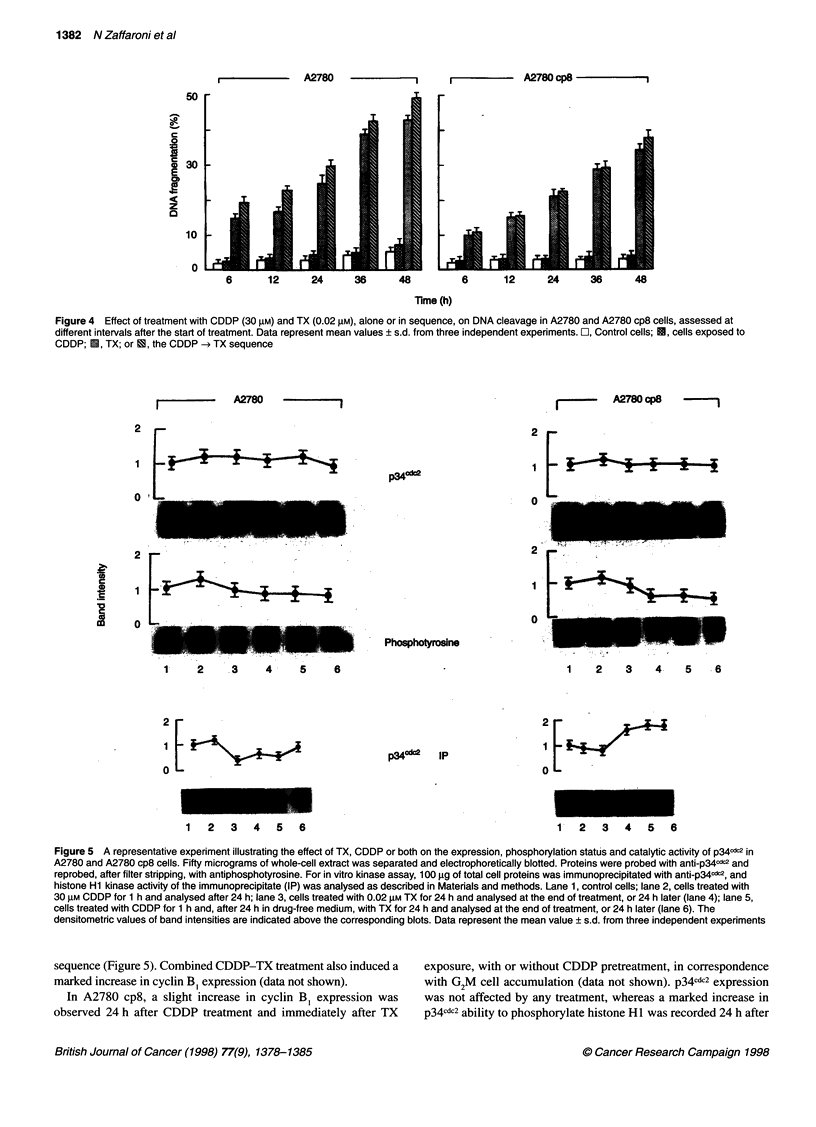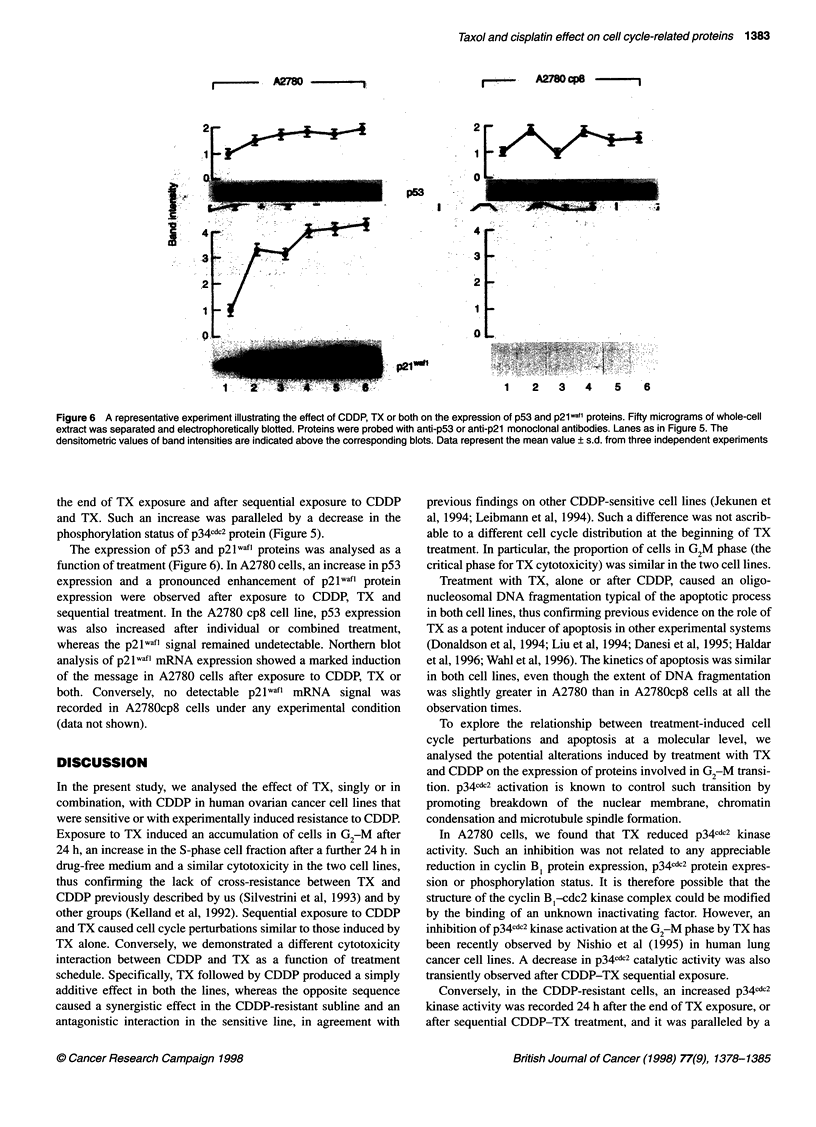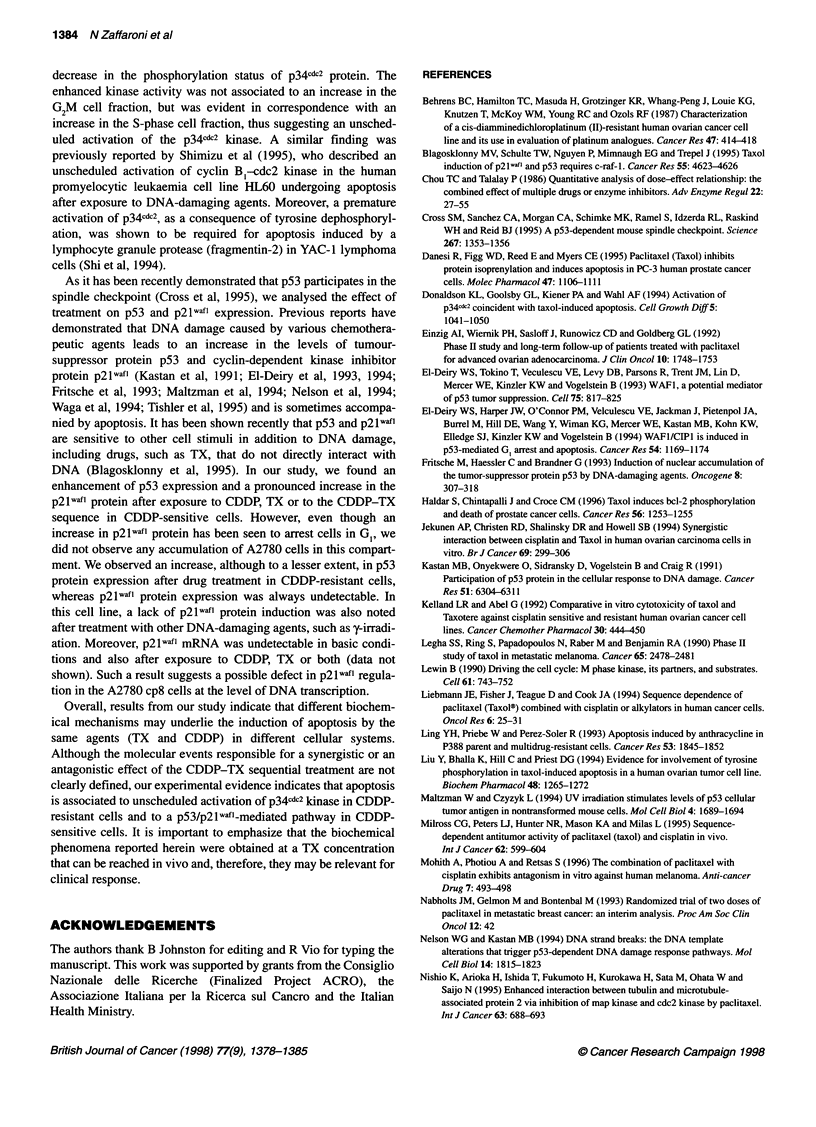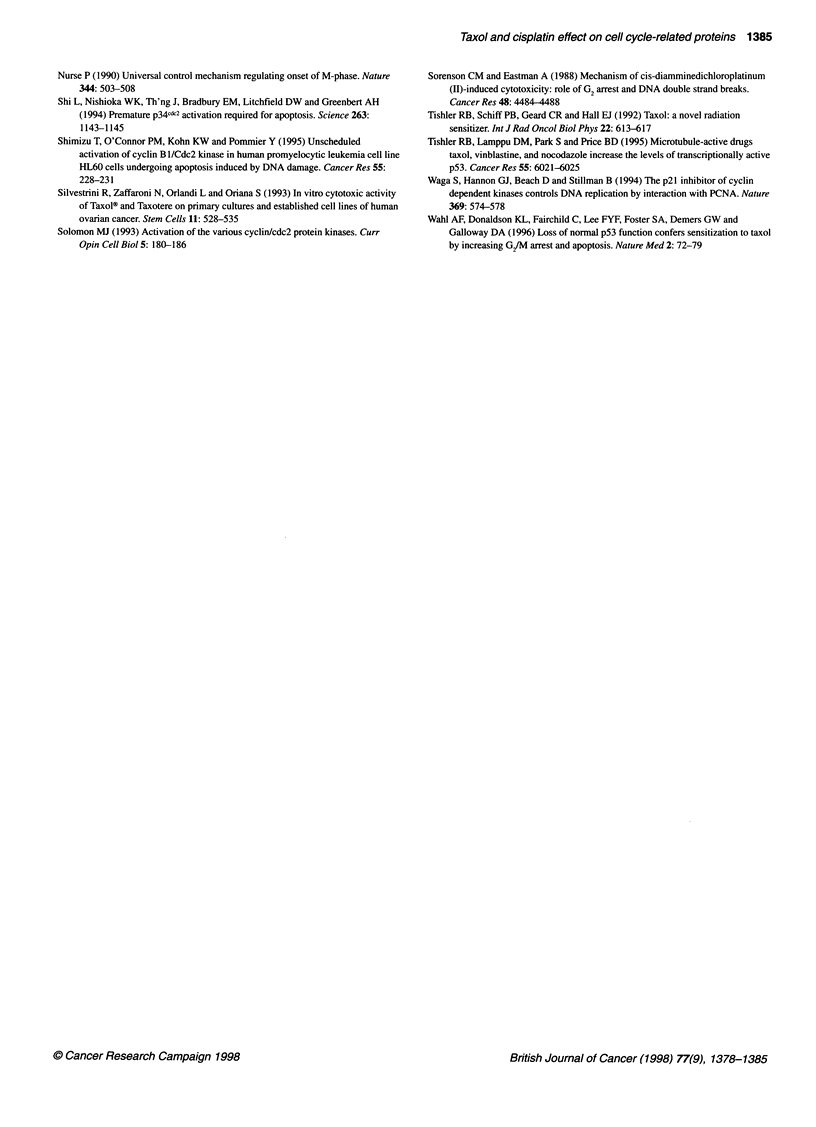# Induction of apoptosis by taxol and cisplatin and effect on cell cycle-related proteins in cisplatin-sensitive and -resistant human ovarian cells.

**DOI:** 10.1038/bjc.1998.230

**Published:** 1998-05

**Authors:** N. Zaffaroni, R. Silvestrini, L. Orlandi, A. Bearzatto, D. Gornati, R. Villa

**Affiliations:** Divisione di Oncologia Sperimentale C, Istituto Nazionale per lo Studio e la Cura dei Tumori, Milan, Italy.

## Abstract

**Images:**


British Journal of Cancer (1998) 77(9), 1378–1385
© 1998 Cancer Research Campaign

### Induction of apoptosis by taxol and cisplatin and effect on cell cycle-related proteins in cisplatin-sensitive and -resistant human ovarian cancer cells

**N Zaffaroni, R Silvestrini, L Orlandi, A Bearzatto, D Gornati and R Villa**

Divisione di Oncologia Sperimentale C, Istituto Nazionale per lo Studio e la Cura dei Tumori, 20133 Milan, Italy

**Summary** The effect of taxol (TX) and cisplatin (CDDP), singly or in association, was assessed on two human ovarian cancer cell lines, one sensitive (A2780) and one resistant (A2780 cp8) to CDDP. Cell lines showed a similar sensitivity to TX, whereas different cytotoxicity results were obtained in the two cell lines as a function of TX and CDDP sequence. Specifically, TX followed by CDDP induced simply additive effects in both cell lines, whereas the opposite sequence produced antagonistic effects in A2780 cells and synergistic effects in A2780 cp8 cells. TX, with or without CDDP, induced oligonucleosomal DNA fragmentation typical of the apoptotic process, but the biochemical mechanisms undergoing apoptosis were different in the two cell lines. In fact, in A2780 cells, TX (with or without CDDP) treatment markedly increased p53 as well as $p21^{waf1}$ protein expression. In A2780 cp8 cells, drug treatment enhanced p53 levels, whereas the expression of $p21^{waf1}$ was always undetectable at mRNA and protein levels. In the latter cell line, a premature activation of $p34^{cdc2}$ kinase was observed in correspondence with the drug-induced increase in the S-phase cell fraction. Such an activation was not ascribable to an increase in the overall expression of $p34^{cdc2}$ or cyclin $B_1$ proteins, but to a dephosphorylation of $p34^{cdc2}$ kinase. Overall, our results indicate that TX-induced apoptosis in human ovarian cancer cells may be sustained by different events at the cell cycle-control level.

**Keywords:** taxol; cisplatin; apoptosis; cell cycle-related proteins; ovarian cancer

Taxol (TX), an antimicrotubule agent that stabilizes mitotic spindles, has a well-documented clinical efficacy in a variety of human neoplasms, including ovarian (Einzig et al, 1992), breast cancer (Nabholts et al, 1993) and cutaneous melanoma (Legha et al, 1990). The encouraging responses as a single agent have prompted the activation of trials of TX in association with cisplatin (CDDP) or doxorubicin. The greatest effect of the CDDP–TX combination in experimental systems was observed when TX preceded the alkylator (Jekunen et al, 1994; Leibmann et al, 1994). However, the type of interaction between the two drugs in the different treatment schedules is controversial (Milross et al, 1995; Mohith et al, 1996), and the biochemical mechanisms responsible for such effects have not been clearly identified.

Preclinical studies on the mechanisms of CDDP–TX activity have been carried out only on CDDP-sensitive cells. In the present study, we analysed the effect of the two-drug combination in a CDDP-resistant ovarian cancer cell line (A2780 cp8) in comparison with that observed in the parental CDDP-sensitive cell line (A2780) to ascertain whether CDDP resistance interferes with the pattern of the CDDP–TX interaction. Moreover, as previous reports have indicated that cell death induced by TX is sustained by an apoptotic process (Donaldson et al, 1994; Liu et al, 1994; Danesi et al, 1995; Haldar et al, 1996; Wahl et al, 1996), we analysed the occurrence of apoptosis after CDDP–TX exposure, and its relation with cell cycle perturbations and the expression of proteins that regulate cell cycle progression. As CDDP and TX are known to induce $G_2M$ phase cell accumulation (Sorenson et al, 1988; Tishler et al, 1992), in our study, we focused on the proteins cyclin $B_1$ and $p34^{cdc2}$ kinase, which control $G_2$–M progression (Lewin, 1990; Nurse, 1990; Salomon et al, 1993). Moreover, based on recent evidence that p53 participates in the spindle checkpoint process, to ensure a correct DNA replication before mitosis (Cross et al, 1995), we also analysed the effect of CDDP–TX treatment on p53 and $p21^{waf1}$ expression.

*Received 7 July 1997*
*Revised 12 September 1997*
*Accepted 27 September 1997*

*Correspondence to:* R Silvestrini, Istituto Nazionale Tumori, Via Venezian 1, 20133 Milano, Italy

### MATERIAL AND METHODS

### Cell lines

A2780 is an ovarian carcinoma cell line derived from an untreated patient. The A2780 cp8 resistant subline was obtained by exposure of the A2780 line to increasing stepwise concentrations of CDDP (Behrens et al, 1987). Cell lines were maintained as a monolayer in RPMI-1640 medium supplemented with 10% fetal calf serum, 2 $\mu$M L-glutamine, 0.25 U $ml^{-1}$ insulin and 0.1% gentamycin. Both cell lines are characterized by a wild-type *p53* gene, as detected by single-strand conformation polymorphism and direct DNA sequencing analyses (data not shown).

### Chemicals

Cisplatin (CDDP, Bristol-Myers, Evansville, IL, USA) was dissolved in 0.9% sodium chloride and protected from light. The drug was diluted with fresh medium immediately before each experiment and used at concentrations of 3–90 $\mu$M. Taxol (TX, Sigma Chemical, St Louis, MO, USA) was stored as a 200 mmol $l^{-1}$ stock solution in dimethyl sulphoxide and then reconstituted and diluted in sterile water to obtain a solvent concentration of less than 0.25%. TX was used at concentrations of 0.01–0.1 μM.

### Cell survival assay

After harvesting in the logarithmic growth phase, cells were seeded in six well plates (45 000 cells per plate; plate surface 9.4 $cm^2$) in 2 ml of fresh medium. Different TX–CDDP combination schedules were tested: (a) 1 h CDDP treatment followed by an incubation for 24 h in drug-free medium, and a final 24 h exposure to TX; (b) 24-h TX treatment followed by a 1-h exposure to CDDP. At the end of treatment, cells were washed with phosphate-buffered saline (PBS) and incubated at 37°C in a 5% carbon dioxide humidified atmosphere for 3 days. Plates were then trypsinized and counted in a particle counter (Coulter Counter model, Coulter Electronics, Luton, UK). The percentages of viable cells were determined by the Trypan blue dye exclusion test. Viability always exceeded 95%. Each experimental sample was run in triplicate. The results were expressed as the cell number of treated samples compared with control samples. In the studies on the cell cycle, cell-cycle related proteins and apoptosis, only scheme a was used.

### Median effect analysis

The approach proposed by Chou and Talalay (1986) was used to determine the nature of the interaction between CDDP and TX. Drugs were always combined at a constant ratio of TX and CDDP concentrations (1:600). The interaction of the two drugs was quantified by determining a combination index (CI) at increasing level of cell kill. CI values of less than or greater than 1 indicate synergy or antagonism, respectively, whereas a CI value of 1 indicates additivity. Each data point represents the mean of at least three experiments, each performed with triplicate cultures.

### Cell cycle distribution analysis

Samples of $1 \times 10^6$ cells were fixed in 70% ethanol. Before analysis, cells were washed in PBS and stained with a solution containing 50 μg $ml^{-1}$ propidium iodide, 50 mg $ml^{-1}$ RNAase, and 0.05% NP40 for 30 min at 4°C. The fluorescence of stained cells was measured using a FACScan flow cytometer (Becton Dickinson, Sunnyvale, CA, USA). A minimum of $1 \times 10^4$ cells was measured for each sample. The percentage of cells in the different cycle phases was evaluated on DNA plots by CellFit software according to the SOBR model (Becton Dickinson).

### Immunoblotting

Cells were lysed on ice with a RIPA buffer (20 mM Tris, pH 7.4, 150 mM sodium chloride, 5 mM sodium fluoride and the protease inhibitors aprotinin, leupeptin and pepstatin at a concentration of 10 μg $ml^{-1}$, and 2 mM phenylmethylsulphonyl fluoride). Each lysate was centrifuged at 15 000 *g* for 20 min, and the protein content of each supernatant was quantified by the Bio-Rad protein assay. Fifty micrograms of total cellular protein was separated on 12% sodium dodecyl sulphate (SDS)–polyacrylamide gel and transferred to nitrocellulose. Filters were blocked overnight in TBS-T buffer (20 mM Tris, 137 mM sodium chloride, pH 7.6, 0.1% Tween 80) with 5% skimmed milk and then incubated with the primary monoclonal antibody anti-$p34^{cdc2}$ (Santa Cruz Biotechnology, Santa Cruz, CA, USA), anti-cyclin $B_1$ (Pharmingen, San Diego, CA, USA), anti-p53 (Oncogene Science, Cambridge, MA, USA) and anti-$p21^{waf1}$ (Oncogene Science). Filters were then incubated with the secondary antibody anti-mouse Ig horseradish peroxidase-linked whole antibody (Amersham, Buckingamshire, UK). Bound antibody was detected using the enhanced chemoluminescence Western blotting detection system (Amersham). To reprobe with alternative antisera, the membranes probed with anti-$p34^{cdc2}$ were immersed in a stripping solution (100 mM 2-mercaptoethanol, 2% SDS, 62.5 mM Tris, pH 6.7) for 30 min at 50°C. Non-specific binding sites were blocked in 5% skimmed milk/TBS-T and the filter reprobed with anti-phosphotyrosine (Boehringer, Mannheim, Germany) as primary antibody.

### Immunoprecipitation and histone H1 kinase assay

Total cellular protein (100 μg) was immunoprecipitated by anti-$p34^{cdc2}$ agarose conjugate (Santa Cruz Biotechnology) for 4 h at 4°C. After washing, immunoprecipitates were resuspended in 50 μl of kinase buffer containing 50 mM Tris, pH 7.4, 10 mM magnesium chloride, 1 mM dithiothreitol and 50 μg $ml^{-1}$ histone H1 (Boehringer Mannheim). After a preincubation of 10 min at 30°C, reactions were started by the addition of 10 μCi of [$\gamma$-$^{32}$P]ATP (specific activity 3000 Ci $mmol^{-1}$), incubated at 30°C for 20 min and stopped by the addition of 50 μl of 2 × SDS gel loading buffer. The mixtures were denatured at 95°C for 5 min and separated on 12% SDS–polyacrylamide gel. Bands were detected by autoradiography and quantified by an Ultrascan XL, enhanced laser densitometer (LKB, Turku, Finland).

### DNA agarose gel electrophoresis

Adherent and floating cells ($3 \times 10^6$) were lysed in a solution containing 10 mM EDTA, 5 mM Tris-HCl (pH 8.0) and 0.5% Triton X-100, for 30 min on ice. Samples were centrifuged, and the supernatant (low-molecular-weight DNA) was separated from the pellet (high-molecular-weight DNA). Both fractions were incubated with 500 units $ml^{-1}$ RNAase A (DNAase free) for 1 h at 37°C and treated with 1% SDS detergent containing 0.5 mg $ml^{-1}$ proteinase K for 3 h at 50°C. The lysate was extracted once with phenol, once with phenol–chloroform–isoamyl alcohol (25:24:1, v/v/v), precipitated with 2.5 volumes of ethanol and 0.1 volume of sodium acetate (3 M, pH 5.2), and then dissolved in TE buffer (10 mM Tris-HCl, pH 7.5, 1 mM EDTA, pH 8.0). Aliquots were electrophoresed in 1.5% agarose gel at 50 V (2.5 V $cm^{-1}$) for 3 h and at 70 V (3.5 V $cm^{-1}$) for 1 h in TBE buffer (89 mM Tris base, 89 mM boric acid, 2 mM EDTA). Gels were stained with 10 μg $ml^{-1}$ ethidium bromide, destained for 20 min in water, and the resultant DNA ladder was visualized and photographed under a UV transilluminator.

### Assessment of DNA cleavage

DNA cleavage in control and treated A2780 and A2780 cp8 cells was quantitatively determined by the method of Ling et al (1993), modified as needed. In brief, exponentially growing cells ($1 \times 10^6$ cells $ml^{-1}$) were prelabelled with 1 μCi [$^{14}$C]thymidine for 24 h and chased for 2 h in fresh medium without thymidine. Labelled cells were exposed to CDDP and TX alone or in sequence and, at different intervals after treatment, adherent and floating cells were collected. Cell pellets were incubated in 0.5 ml of hypotonic lysis buffer, containing 10 mM Tris-HCl (pH 8.0),

© Cancer Research Campaign 1998

**Figure 1** Dose–response growth curves of A2780 (●) and A2780 cp8 cells (■) treated with TX for 24 h (**A**) or with CDDP for 1 h (**B**). Data represent mean values ± s.d. from three independent experiments

**Figure 2** Combination index plots for the interaction between CDDP and TX when given according to the following sequences: TX for 24 h followed by CDDP for 1 h (**A**) or CDDP for 1 h and, after 24 h in drug-free medium, TX for 24 h (**B**) in A2780 (●) and A2780 cp8 (■) cells. Data represent mean values ± s.d. from three independent experiments

1 mM EDTA and 0.2% Triton x-100 at room temperature for 30 min, and centrifuged at 12 000 *g* for 30 min. The radioactivity in the supernatant (detergent-soluble, low-molecular-weight DNA) and in the pellet (intact chromatin DNA) was determined with a liquid scintillation counter. The percentage of DNA fragmentation was calculated using the following formula:

$$\text{\% DNA fragmentation} = \frac{\text{c.p.m. in supernatant}}{\text{c.p.m. in supernatant} + \text{c.p.m. in pellet}}$$

### RESULTS

Sensitivity to a 1-h CDDP exposure was markedly different for A2780 and A2780 cp8, and concentrations inhibiting cell proliferation by 50% ($IC_{50}$) were 25 μM and 60 μM respectively (Figure 1). Conversely, a similar dose-dependent effect of a 24-h exposure to TX was observed in the two cell lines, with an $IC_{50}$ of 0.016 μM and 0.018 μM in A2780 and A2780 cp8 respectively (Figure 1).

Figure 2 shows the plots of the CI for the interaction between CDDP and TX as a function of treatment sequence for A2780 and A2780 cp8. A 24-h TX treatment followed by a 1-h CDDP exposure consistently produced simply additive effects, as indicated by CI values very close to 1 (Figure 2A). Conversely, the inverse sequence (i.e. CDDP followed by TX) caused markedly different cytotoxic effects in the two cell lines. Specifically, an antagonistic interaction was observed in CDDP-sensitive cells, whereas a clear synergistic effect was evident in the CDDP-resistant subline. The magnitude of the synergy was almost constant at the different levels of cell kill (Figure 2B).

DNA flow cytometric analysis was performed to determine whether cell cycle perturbations could be responsible for the different CDDP–TX interaction patterns (Table 1). A transient $G_2M$ block was observed in both cell lines 24 h after a 1-h exposure to CDDP. Similarly, an accumulation of cells in the $G_2M$ phase after a 24-h treatment with TX and an increase in the S-phase cell fraction after an additional 24-h in drug-free medium were observed in both cell lines. At the same time, a slight accumulation in the $G_2M$ compartment was still appreciable only in CDDP-sensitive cells. The CDDP–TX sequential treatment induced cell cycle perturbations similar to those caused by TX alone.

© Cancer Research Campaign 1998

**Table 1** Cell cycle perturbations induced by CDDP and TX

| | Time | | | | | | | | |
|---|---|---|---|---|---|---|---|---|---|
| | 24 h | | | 48 h | | | 72 h | | |
| | $G_{0/1}$ | S | $G_2M$ | $G_{0/1}$ | S | $G_2M$ | $G_{0/1}$ | S | $G_2M$ |
| *A2780* | | | | | | | | | |
| Control | 40±3 | 44±4 | 16±5 | 45±5 | 39±3 | 16±4 | 64±4 | 26±3 | 10±5 |
| CDDP[a] (1 h) | 26±4 | 36±3 | 38±5 | 46±5 | 35±4 | 19±3 | 49±6 | 37±4 | 14±3 |
| 24 h[b] → TX[c] (24 h) | – | – | – | 22±3 | 28±4 | 50±7 | 27±5 | 49±6 | 24±3 |
| CDDP[a] (1 h) → 24 h[b] → TX[c] (24 h) | – | – | – | 9±3 | 40±3 | 51±5 | 21±3 | 53±6 | 26±4 |
| *A2780cp8* | | | | | | | | | |
| Control | 37±4 | 48±3 | 15±5 | 42±7 | 43±5 | 15±6 | 53±3 | 36±4 | 11±5 |
| CDDP[a] (1 h) | 29±3 | 39±5 | 32±5 | 40±5 | 41±6 | 19±4 | 43±5 | 39±6 | 14±6 |
| 24 h[b] → TX[c] (24 h) | – | – | – | 5±2 | 33±6 | 62±7 | 17±4 | 71±8 | 12±3 |
| CDDP[a] (1 h) → 24 h[b] → TX[c] (24 h) | – | – | – | 8±3 | 51±4 | 40±3 | 16±4 | 79±4 | 5±5 |

[a]30 μM. [b]24 h in drug-free medium. [c]0.02 μM. Data represent mean values ± s.d. from three independent experiments.

**Figure 3** Electrophoretic pattern of DNA extracted from A2780 and A2780 cp8 cells treated with CDDP, TX or both. Lane 1, lambda DNA Bste II molecular weight markers; lane 2, control cells; lane 3, cells treated with 30 μM CDDP for 1 h and analysed after 24 h; lane 4, cells treated with 0.02 μM TX for 24 h and analysed at the end of treatment, or 24 h later (lane 5); lane 6, cells treated with CDDP for 1 h and, after 24 h in drug-free medium, with TX for 24 h and analysed at the end of treatment, or 24 h later (lane 7)

Gel electrophoresis analysis of DNA from CDDP-sensitive and CDDP-resistant floating cells (Figure 3) showed accumulation of oligonucleosome fragments at the end of a 24-h exposure to TX and after an additional 24 h in drug-free medium. Similarly, DNA fragmentation was recorded after exposure to the CDDP–TX sequence, whereas no DNA cleavage was observed after exposure to CDDP alone. Adherent cells never showed oligonucleosomal fragmentation independently of drug treatment (data not shown). The quantitative assessment of DNA degradation was performed on [$^{14}$C]-thymidine-labelled A2780 and A2780 cp8 cells, at different intervals after individual or combined treatment (Figure 4). A negligible DNA fragmentation, similar to that observed in untreated cells, was observed in cells exposed to CDDP, whereas a marked DNA cleavage was observed, to a similar extent, in cells treated with TX alone or with the TX–CDDP sequence. The amount of fragmented DNA progressively increased with time in both cell lines. However, the extent of DNA fragmentation was consistently greater in A2780 than in A2780 cp8 cells at all the observation times.

The expression of proteins involved in $G_2$–M transition was analysed before and after TX and CDDP exposure. In A2780 cells, a slight increase in cyclin $B_1$ expression was observed 24 h after CDDP exposure in correspondence with $G_2M$ accumulation (data not shown). No appreciable difference with respect to controls was recorded in p34$^{cdc2}$ expression nor with respect to the ability to phosphorylate histone H1 (Figure 5). TX treatment induced a reduction in p34$^{cdc2}$ kinase activity at all the observation times, without any appreciable inhibition of cyclin $B_1$ or p34$^{cdc2}$ protein expression, as well as variations in the phosphorylation status of the latter protein (Figure 5). A decrease in p34$^{cdc2}$ catalytic activity was also transiently observed immediately after exposure to the CDDP–TX

*© Cancer Research Campaign 1998*

**Figure 4** Effect of treatment with CDDP (30 μM) and TX (0.02 μM), alone or in sequence, on DNA cleavage in A2780 and A2780 cp8 cells, assessed at different intervals after the start of treatment. Data represent mean values ± s.d. from three independent experiments. □, Control cells; ▩, cells exposed to CDDP; ▩, TX; or ▧, the CDDP → TX sequence

**Figure 5** A representative experiment illustrating the effect of TX, CDDP or both on the expression, phosphorylation status and catalytic activity of $p34^{cdc2}$ in A2780 and A2780 cp8 cells. Fifty micrograms of whole-cell extract was separated and electrophoretically blotted. Proteins were probed with anti-$p34^{cdc2}$ and reprobed, after filter stripping, with antiphosphotyrosine. For in vitro kinase assay, 100 μg of total cell proteins was immunoprecipitated with anti-$p34^{cdc2}$, and histone H1 kinase activity of the immunoprecipitate (IP) was analysed as described in Materials and methods. Lane 1, control cells; lane 2, cells treated with 30 μM CDDP for 1 h and analysed after 24 h; lane 3, cells treated with 0.02 μM TX for 24 h and analysed at the end of treatment, or 24 h later (lane 4); lane 5, cells treated with CDDP for 1 h and, after 24 h in drug-free medium, with TX for 24 h and analysed at the end of treatment, or 24 h later (lane 6). The densitometric values of band intensities are indicated above the corresponding blots. Data represent the mean value ± s.d. from three independent experiments sequence (Figure 5). Combined CDDP–TX treatment also induced a marked increase in cyclin $B_1$ expression (data not shown).

In A2780 cp8, a slight increase in cyclin $B_1$ expression was observed 24 h after CDDP treatment and immediately after TX exposure, with or without CDDP pretreatment, in correspondence with $G_2M$ cell accumulation (data not shown). $p34^{cdc2}$ expression was not affected by any treatment, whereas a marked increase in $p34^{cdc2}$ ability to phosphorylate histone H1 was recorded 24 h after

© Cancer Research Campaign 1998

**Figure 6** A representative experiment illustrating the effect of CDDP, TX or both on the expression of p53 and p21$^{waf1}$ proteins. Fifty micrograms of whole-cell extract was separated and electrophoretically blotted. Proteins were probed with anti-p53 or anti-p21 monoclonal antibodies. Lanes as in Figure 5. The densitometric values of band intensities are indicated above the corresponding blots. Data represent the mean value ± s.d. from three independent experiments the end of TX exposure and after sequential exposure to CDDP and TX. Such an increase was paralleled by a decrease in the phosphorylation status of p34$^{cdc2}$ protein (Figure 5).

The expression of p53 and p21$^{waf1}$ proteins was analysed as a function of treatment (Figure 6). In A2780 cells, an increase in p53 expression and a pronounced enhancement of p21$^{waf1}$ protein expression were observed after exposure to CDDP, TX and sequential treatment. In the A2780 cp8 cell line, p53 expression was also increased after individual or combined treatment, whereas the p21$^{waf1}$ signal remained undetectable. Northern blot analysis of p21$^{waf1}$ mRNA expression showed a marked induction of the message in A2780 cells after exposure to CDDP, TX or both. Conversely, no detectable p21$^{waf1}$ mRNA signal was recorded in A2780cp8 cells under any experimental condition (data not shown).

### DISCUSSION

In the present study, we analysed the effect of TX, singly or in combination, with CDDP in human ovarian cancer cell lines that were sensitive or with experimentally induced resistance to CDDP. Exposure to TX induced an accumulation of cells in $G_2$–M after 24 h, an increase in the S-phase cell fraction after a further 24 h in drug-free medium and a similar cytotoxicity in the two cell lines, thus confirming the lack of cross-resistance between TX and CDDP previously described by us (Silvestrini et al, 1993) and by other groups (Kelland et al, 1992). Sequential exposure to CDDP and TX caused cell cycle perturbations similar to those induced by TX alone. Conversely, we demonstrated a different cytotoxicity interaction between CDDP and TX as a function of treatment schedule. Specifically, TX followed by CDDP produced a simply additive effect in both the lines, whereas the opposite sequence caused a synergistic effect in the CDDP-resistant subline and an antagonistic interaction in the sensitive line, in agreement with previous findings on other CDDP-sensitive cell lines (Jekunen et al, 1994; Leibmann et al, 1994). Such a difference was not ascribable to a different cell cycle distribution at the beginning of TX treatment. In particular, the proportion of cells in $G_2$M phase (the critical phase for TX cytotoxicity) was similar in the two cell lines.

Treatment with TX, alone or after CDDP, caused an oligonucleosomal DNA fragmentation typical of the apoptotic process in both cell lines, thus confirming previous evidence on the role of TX as a potent inducer of apoptosis in other experimental systems (Donaldson et al, 1994; Liu et al, 1994; Danesi et al, 1995; Haldar et al, 1996; Wahl et al, 1996). The kinetics of apoptosis was similar in both cell lines, even though the extent of DNA fragmentation was slightly greater in A2780 than in A2780cp8 cells at all the observation times.

To explore the relationship between treatment-induced cell cycle perturbations and apoptosis at a molecular level, we analysed the potential alterations induced by treatment with TX and CDDP on the expression of proteins involved in $G_2$–M transition. p34$^{cdc2}$ activation is known to control such transition by promoting breakdown of the nuclear membrane, chromatin condensation and microtubule spindle formation.

In A2780 cells, we found that TX reduced p34$^{cdc2}$ kinase activity. Such an inhibition was not related to any appreciable reduction in cyclin $B_1$ protein expression, p34$^{cdc2}$ protein expression or phosphorylation status. It is therefore possible that the structure of the cyclin $B_1$–cdc2 kinase complex could be modified by the binding of an unknown inactivating factor. However, an inhibition of p34$^{cdc2}$ kinase activation at the $G_2$–M phase by TX has been recently observed by Nishio et al (1995) in human lung cancer cell lines. A decrease in p34$^{cdc2}$ catalytic activity was also transiently observed after CDDP–TX sequential exposure.

Conversely, in the CDDP-resistant cells, an increased p34$^{cdc2}$ kinase activity was recorded 24 h after the end of TX exposure, or after sequential CDDP–TX treatment, and it was paralleled by a

© Cancer Research Campaign 1998 

decrease in the phosphorylation status of p34[cdc2] protein. The enhanced kinase activity was not associated to an increase in the $G_2M$ cell fraction, but was evident in correspondence with an increase in the S-phase cell fraction, thus suggesting an unscheduled activation of the p34[cdc2] kinase. A similar finding was previously reported by Shimizu et al (1995), who described an unscheduled activation of cyclin $B_1$–cdc2 kinase in the human promyelocytic leukaemia cell line HL60 undergoing apoptosis after exposure to DNA-damaging agents. Moreover, a premature activation of p34[cdc2], as a consequence of tyrosine dephosphorylation, was shown to be required for apoptosis induced by a lymphocyte granule protease (fragmentin-2) in YAC-1 lymphoma cells (Shi et al, 1994).

As it has been recently demonstrated that p53 participates in the spindle checkpoint (Cross et al, 1995), we analysed the effect of treatment on p53 and p21[waf1] expression. Previous reports have demonstrated that DNA damage caused by various chemotherapeutic agents leads to an increase in the levels of tumour-suppressor protein p53 and cyclin-dependent kinase inhibitor protein p21[waf1] (Kastan et al, 1991; El-Deiry et al, 1993, 1994; Fritsche et al, 1993; Maltzman et al, 1994; Nelson et al, 1994; Waga et al, 1994; Tishler et al, 1995) and is sometimes accompanied by apoptosis. It has been shown recently that p53 and p21[waf1] are sensitive to other cell stimuli in addition to DNA damage, including drugs, such as TX, that do not directly interact with DNA (Blagosklonny et al, 1995). In our study, we found an enhancement of p53 expression and a pronounced increase in the p21[waf1] protein after exposure to CDDP, TX or to the CDDP–TX sequence in CDDP-sensitive cells. However, even though an increase in p21[waf1] protein has been seen to arrest cells in $G_1$, we did not observe any accumulation of A2780 cells in this compartment. We observed an increase, although to a lesser extent, in p53 protein expression after drug treatment in CDDP-resistant cells, whereas p21[waf1] protein expression was always undetectable. In this cell line, a lack of p21[waf1] protein induction was also noted after treatment with other DNA-damaging agents, such as $\gamma$-irradiation. Moreover, p21[waf1] mRNA was undetectable in basic conditions and also after exposure to CDDP, TX or both (data not shown). Such a result suggests a possible defect in p21[waf1] regulation in the A2780 cp8 cells at the level of DNA transcription.

Overall, results from our study indicate that different biochemical mechanisms may underlie the induction of apoptosis by the same agents (TX and CDDP) in different cellular systems. Although the molecular events responsible for a synergistic or an antagonistic effect of the CDDP–TX sequential treatment are not clearly defined, our experimental evidence indicates that apoptosis is associated to unscheduled activation of p34[cdc2] kinase in CDDP-resistant cells and to a p53/p21[waf1]-mediated pathway in CDDP-sensitive cells. It is important to emphasize that the biochemical phenomena reported herein were obtained at a TX concentration that can be reached in vivo and, therefore, they may be relevant for clinical response.

### ACKNOWLEDGEMENTS

The authors thank B Johnston for editing and R Vio for typing the manuscript. This work was supported by grants from the Consiglio Nazionale delle Ricerche (Finalized Project ACRO), the Associazione Italiana per la Ricerca sul Cancro and the Italian Health Ministry.

### REFERENCES

Behrens BC, Hamilton TC, Masuda H, Grotzinger KR, Whang-Peng J, Louie KG, Knutzen T, McKoy WM, Young RC and Ozols RF (1987) Characterization of a cis-diamminedichloroplatinum (II)-resistant human ovarian cancer cell line and its use in evaluation of platinum analogues. *Cancer Res* **47**: 414–418

Blagosklonny MV, Schulte TW, Nguyen P, Mimnaugh EG and Trepel J (1995) Taxol induction of p21[waf1] and p53 requires c-raf-1. *Cancer Res* **55**: 4623–4626

Chou TC and Talalay P (1986) Quantitative analysis of dose–effect relationship: the combined effect of multiple drugs or enzyme inhibitors. *Adv Enzyme Regul* **22**: 27–55

Cross SM, Sanchez CA, Morgan CA, Schimke MK, Ramel S, Idzerda RL, Raskind WH and Reid BJ (1995) A p53-dependent mouse spindle checkpoint. *Science* **267**: 1353–1356

Danesi R, Figg WD, Reed E and Myers CE (1995) Paclitaxel (Taxol) inhibits protein isoprenylation and induces apoptosis in PC-3 human prostate cancer cells. *Molec Pharmacol* **47**: 1106–1111

Donaldson KL, Goolsby GL, Kiener PA and Wahl AF (1994) Activation of p34[cdc2] coincident with taxol-induced apoptosis. *Cell Growth Diff* **5**: 1041–1050

Einzig AI, Wiernik PH, Sasloff J, Runowicz CD and Goldberg GL (1992) Phase II study and long-term follow-up of patients treated with paclitaxel for advanced ovarian adenocarcinoma. *J Clin Oncol* **10**: 1748–1753

El-Deiry WS, Tokino T, Veculescu VE, Levy DB, Parsons R, Trent JM, Lin D, Mercer WE, Kinzler KW and Vogelstein B (1993) WAF1, a potential mediator of p53 tumor suppression. *Cell* **75**: 817–825

El-Deiry WS, Harper JW, O'Connor PM, Velculescu VE, Jackman J, Pietenpol JA, Burrel M, Hill DE, Wang Y, Wiman KG, Mercer WE, Kastan MB, Kohn KW, Elledge SJ, Kinzler KW and Vogelstein B (1994) WAF1/CIP1 is induced in p53-mediated $G_1$ arrest and apoptosis. *Cancer Res* **54**: 1169–1174

Fritsche M, Haessler C and Brandner G (1993) Induction of nuclear accumulation of the tumor-suppressor protein p53 by DNA-damaging agents. *Oncogene* **8**: 307–318

Haldar S, Chintapalli J and Croce CM (1996) Taxol induces bcl-2 phosphorylation and death of prostate cancer cells. *Cancer Res* **56**: 1253–1255

Jekunen AP, Christen RD, Shalinsky DR and Howell SB (1994) Synergistic interaction between cisplatin and Taxol in human ovarian carcinoma cells in vitro. *Br J Cancer* **69**: 299–306

Kastan MB, Onyekwere O, Sidransky D, Vogelstein B and Craig R (1991) Participation of p53 protein in the cellular response to DNA damage. *Cancer Res* **51**: 6304–6311

Kelland LR and Abel G (1992) Comparative in vitro cytotoxicity of taxol and Taxotere against cisplatin sensitive and resistant human ovarian cancer cell lines. *Cancer Chemother Pharmacol* **30**: 444–450

Legha SS, Ring S, Papadopoulos N, Raber M and Benjamin RA (1990) Phase II study of taxol in metastatic melanoma. *Cancer* **65**: 2478–2481

Lewin B (1990) Driving the cell cycle: M phase kinase, its partners, and substrates. *Cell* **61**: 743–752

Liebmann JE, Fisher J, Teague D and Cook JA (1994) Sequence dependence of paclitaxel (Taxol®) combined with cisplatin or alkylators in human cancer cells. *Oncol Res* **6**: 25–31

Ling YH, Priebe W and Perez-Soler R (1993) Apoptosis induced by anthracycline in P388 parent and multidrug-resistant cells. *Cancer Res* **53**: 1845–1852

Liu Y, Bhalla K, Hill C and Priest DG (1994) Evidence for involvement of tyrosine phosphorylation in taxol-induced apoptosis in a human ovarian tumor cell line. *Biochem Pharmacol* **48**: 1265–1272

Maltzman W and Czyzyk L (1994) UV irradiation stimulates levels of p53 cellular tumor antigen in nontransformed mouse cells. *Mol Cell Biol* **4**: 1689–1694

Milross CG, Peters LJ, Hunter NR, Mason KA and Milas L (1995) Sequence-dependent antitumor activity of paclitaxel (taxol) and cisplatin in vivo. *Int J Cancer* **62**: 599–604

Mohith A, Photiou A and Retsas S (1996) The combination of paclitaxel with cisplatin exhibits antagonism in vitro against human melanoma. *Anti-cancer Drug* **7**: 493–498

Nabholts JM, Gelmon M and Bontenbal M (1993) Randomized trial of two doses of paclitaxel in metastatic breast cancer: an interim analysis. *Proc Am Soc Clin Oncol* **12**: 42

Nelson WG and Kastan MB (1994) DNA strand breaks: the DNA template alterations that trigger p53-dependent DNA damage response pathways. *Mol Cell Biol* **14**: 1815–1823

Nishio K, Arioka H, Ishida T, Fukumoto H, Kurokawa H, Sata M, Ohata W and Saijo N (1995) Enhanced interaction between tubulin and microtubule-associated protein 2 via inhibition of map kinase and cdc2 kinase by paclitaxel. *Int J Cancer* **63**: 688–693

© Cancer Research Campaign 1998

Nurse P (1990) Universal control mechanism regulating onset of M-phase. *Nature* **344**: 503–508

Shi L, Nishioka WK, Th'ng J, Bradbury EM, Litchfield DW and Greenbert AH (1994) Premature p34$^{cdc2}$ activation required for apoptosis. *Science* **263**: 1143–1145

Shimizu T, O'Connor PM, Kohn KW and Pommier Y (1995) Unscheduled activation of cyclin B1/Cdc2 kinase in human promyelocytic leukemia cell line HL60 cells undergoing apoptosis induced by DNA damage. *Cancer Res* **55**: 228–231

Silvestrini R, Zaffaroni N, Orlandi L and Oriana S (1993) In vitro cytotoxic activity of Taxol® and Taxotere on primary cultures and established cell lines of human ovarian cancer. *Stem Cells* **11**: 528–535

Solomon MJ (1993) Activation of the various cyclin/cdc2 protein kinases. *Curr Opin Cell Biol* **5**: 180–186

Sorenson CM and Eastman A (1988) Mechanism of cis-diamminedichloroplatinum (II)-induced cytotoxicity: role of $G_2$ arrest and DNA double strand breaks. *Cancer Res* **48**: 4484–4488

Tishler RB, Schiff PB, Geard CR and Hall EJ (1992) Taxol: a novel radiation sensitizer. *Int J Rad Oncol Biol Phys* **22**: 613–617

Tishler RB, Lamppu DM, Park S and Price BD (1995) Microtubule-active drugs taxol, vinblastine, and nocodazole increase the levels of transcriptionally active p53. *Cancer Res* **55**: 6021–6025

Waga S, Hannon GJ, Beach D and Stillman B (1994) The p21 inhibitor of cyclin dependent kinases controls DNA replication by interaction with PCNA. *Nature* **369**: 574–578

Wahl AF, Donaldson KL, Fairchild C, Lee FYF, Foster SA, Demers GW and Galloway DA (1996) Loss of normal p53 function confers sensitization to taxol by increasing $G_2$/M arrest and apoptosis. *Nature Med* **2**: 72–79

© Cancer Research Campaign 1998